# Supplementary material for: Sweet and sticky: increased cell adhesion through click-mediated functionalization of regenerative liver progenitor cells
Source: Commun Biol. 2025 Jul 10;8:1023. doi: 10.1038/s42003-025-08408-x (PMC12246442; doi:10.1038/s42003-025-08408-x)
Supplement: Supplementary file 2 — Supplementary Information [file 42003_2025_8408_MOESM2_ESM.pdf]

Supporting Information

Sweet and sticky: increased cell adhesion through click-mediated functionalization of regenerative liver progenitor cells

Amaziah R. Alipio[1], Melissa R. Vieira[2,3], Tamara Haefeli[4], Lisa Hoelting[4], Olivier Frey[4], Alicia J. El Haj[2,3], Maria C. Arno[1,5]**

[1] School of Chemistry, University of Birmingham, Edgbaston, Birmingham, B15 2TT, United Kingdom

[2] Healthcare Technologies Institute, Institute of Translational Medicine, University of Birmingham, Edgbaston, Birmingham, B15 2TH, United Kingdom

[3] School of Chemical Engineering, University of Birmingham, Edgbaston, Birmingham, B15 2TH, United Kingdom

[4] InSphero AG, Wagistrasse 27A, 8952, Schlieren, Switzerland

[5] Institute of Cancer and Genomic Sciences, University of Birmingham, Edgbaston, Birmingham, B15 2TT, United Kingdom

m.c.arno@bham.ac.uk

a.elhaj@bham.ac.uk

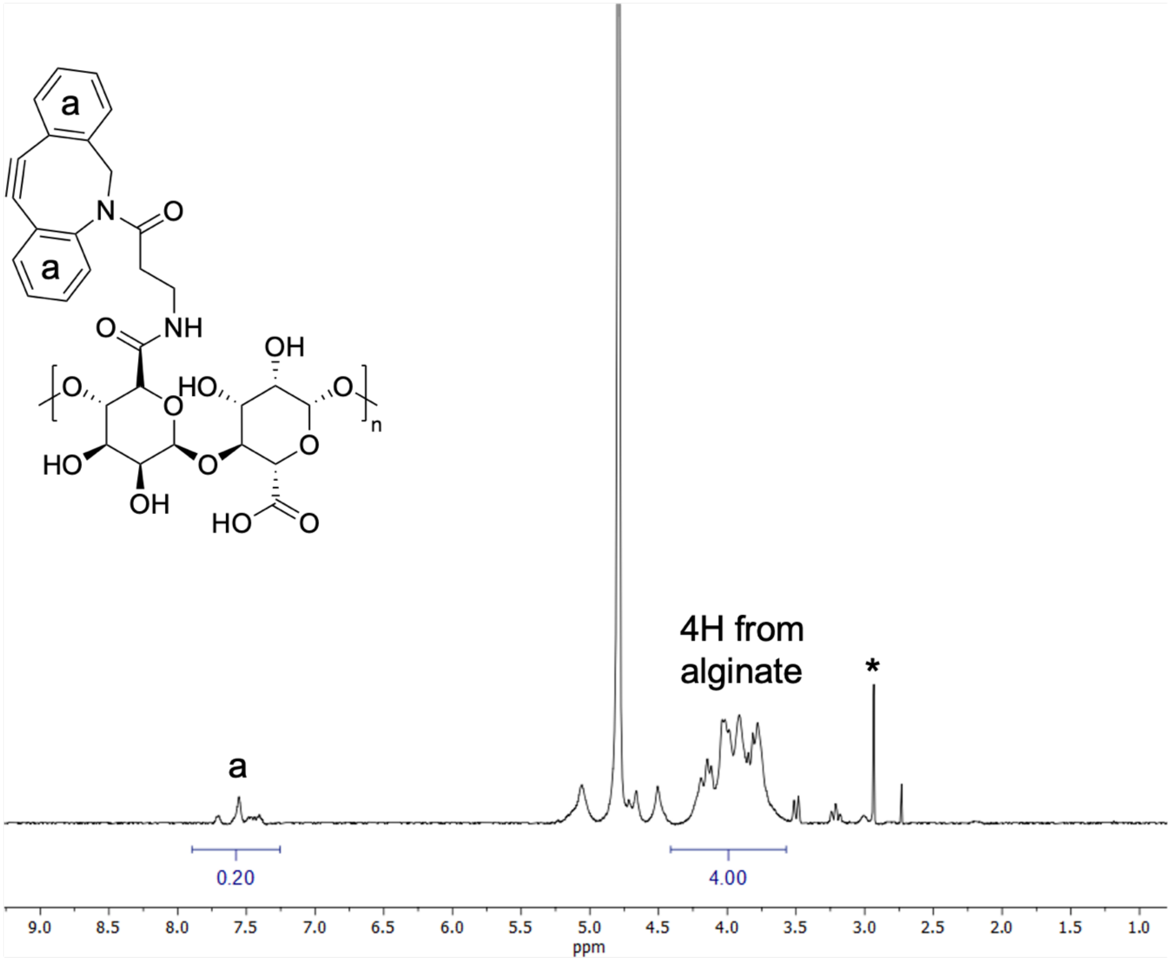

Supplementary Fig. 1. ^{1}H NMR spectrum (400 MHz, D_2O) of alginate functionalized with DBCO (Alg4). * = DMSO. Integration of the aromatic region for DBCO protons indicates a 4% functionalization (0.20 proton/8 = 0.025 units of DBCO per repeating unit, hence 5 in total).

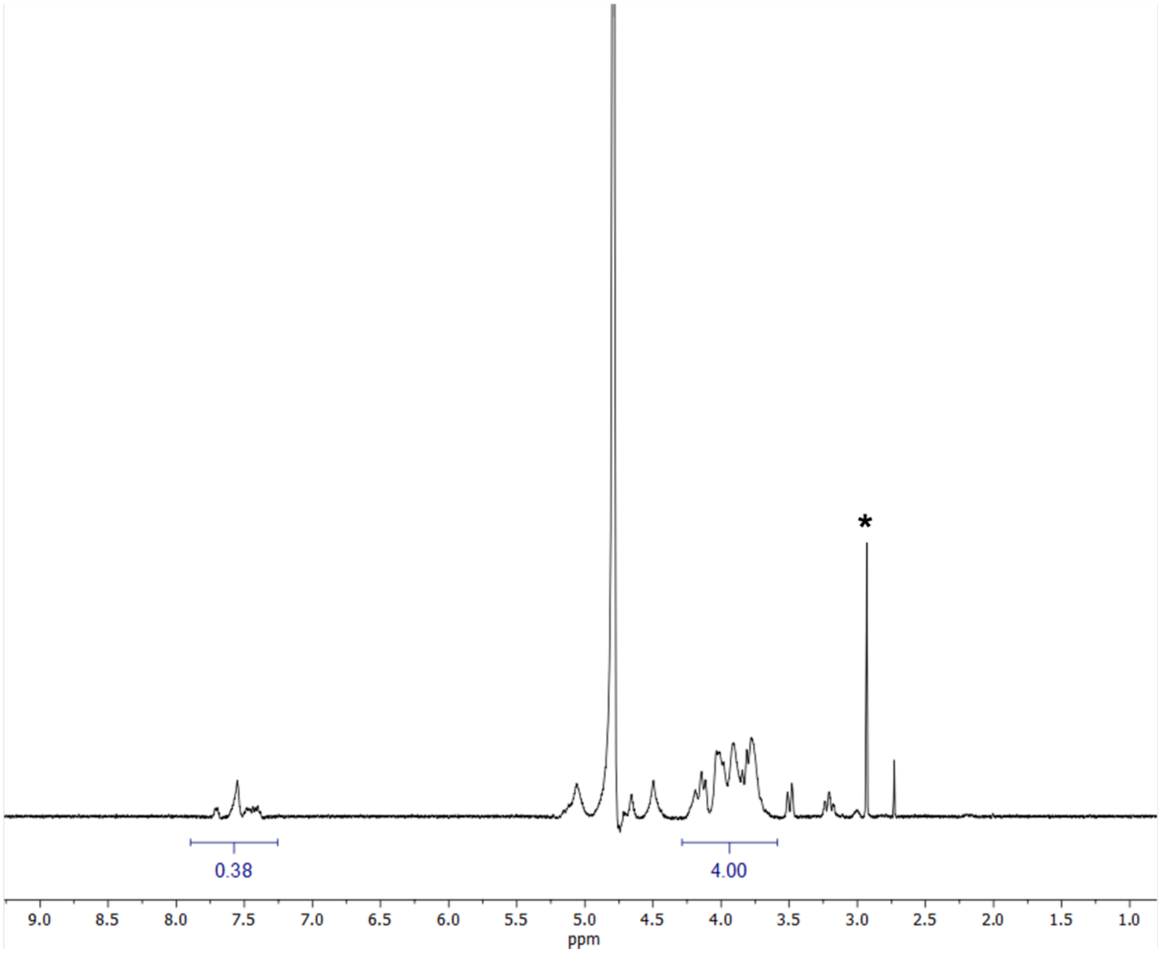

Supplementary Fig. 2. ^1H NMR spectrum (400 MHz, D_2O) of alginate functionalized with DBCO (Alg8). * = DMSO. Integration of the aromatic region for DBCO protons indicates an 8% functionalization (0.38 proton/8 = 0.05 units of DBCO per repeating unit, hence 10 in total).

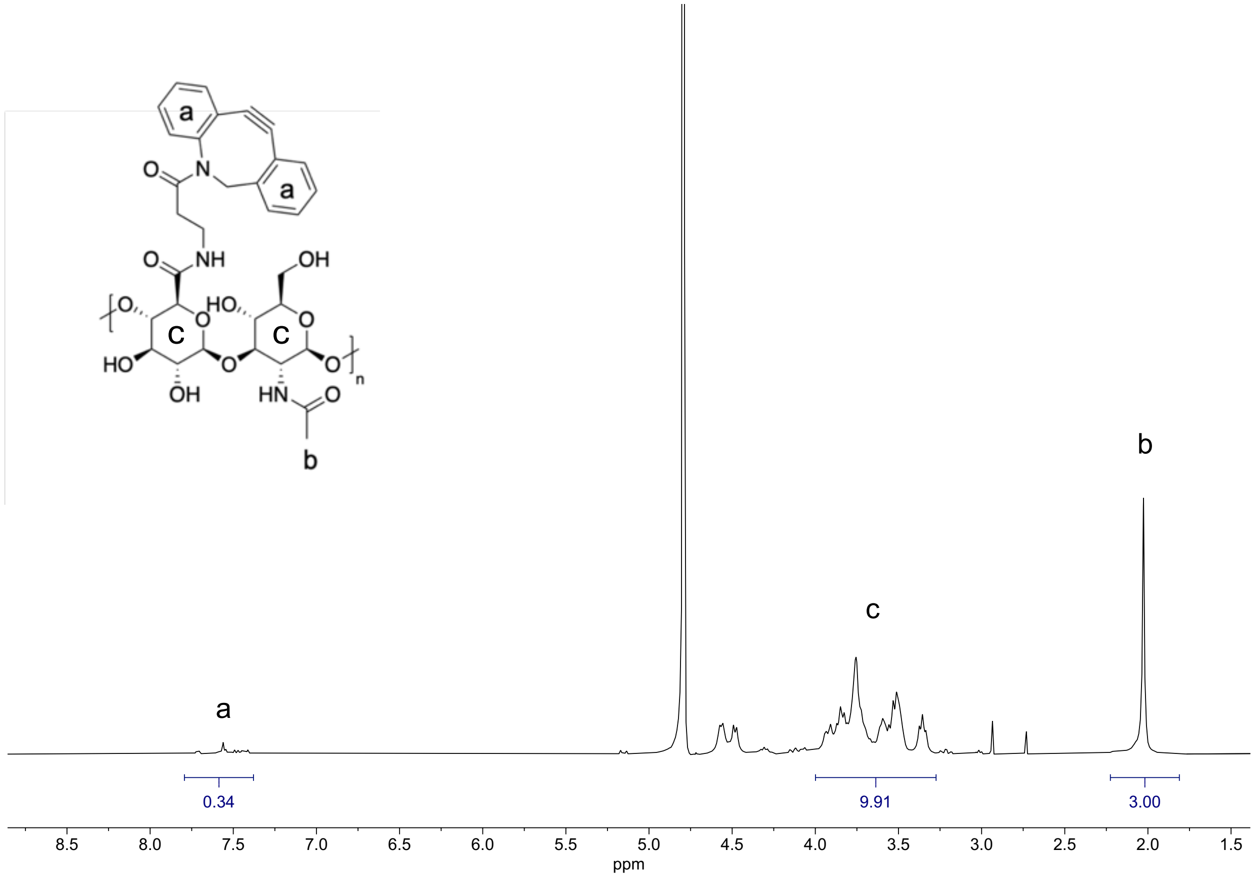

Supplementary Fig. 3. ^{1}H NMR spectrum (400 MHz, D_2O) of hyaluronic acid functionalized with DBCO (HA4). * = DMSO. Integration of the aromatic region for DBCO protons indicates a 4% functionalization (0.34 proton/8 = 0.04 units of DBCO per repeating unit, hence 5 in total).

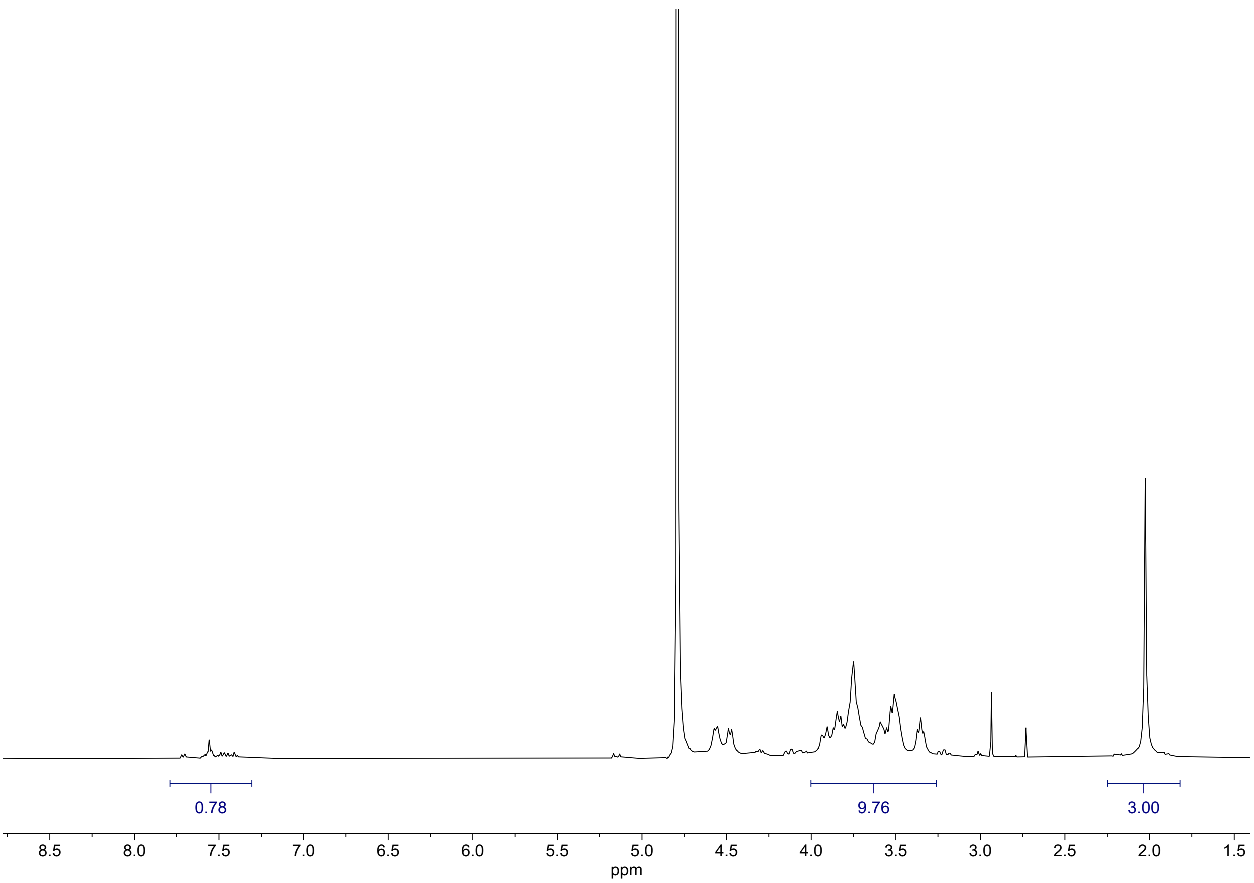

Supplementary Fig. 4. ^{1}H NMR spectrum (400 MHz, D_2O) of hyaluronic acid functionalized with DBCO (HA8). * = DMSO. Integration of the aromatic region for DBCO protons indicates an 8% functionalization (0.78 proton/8 = 0.1 units of DBCO per repeating unit, hence 11 in total).

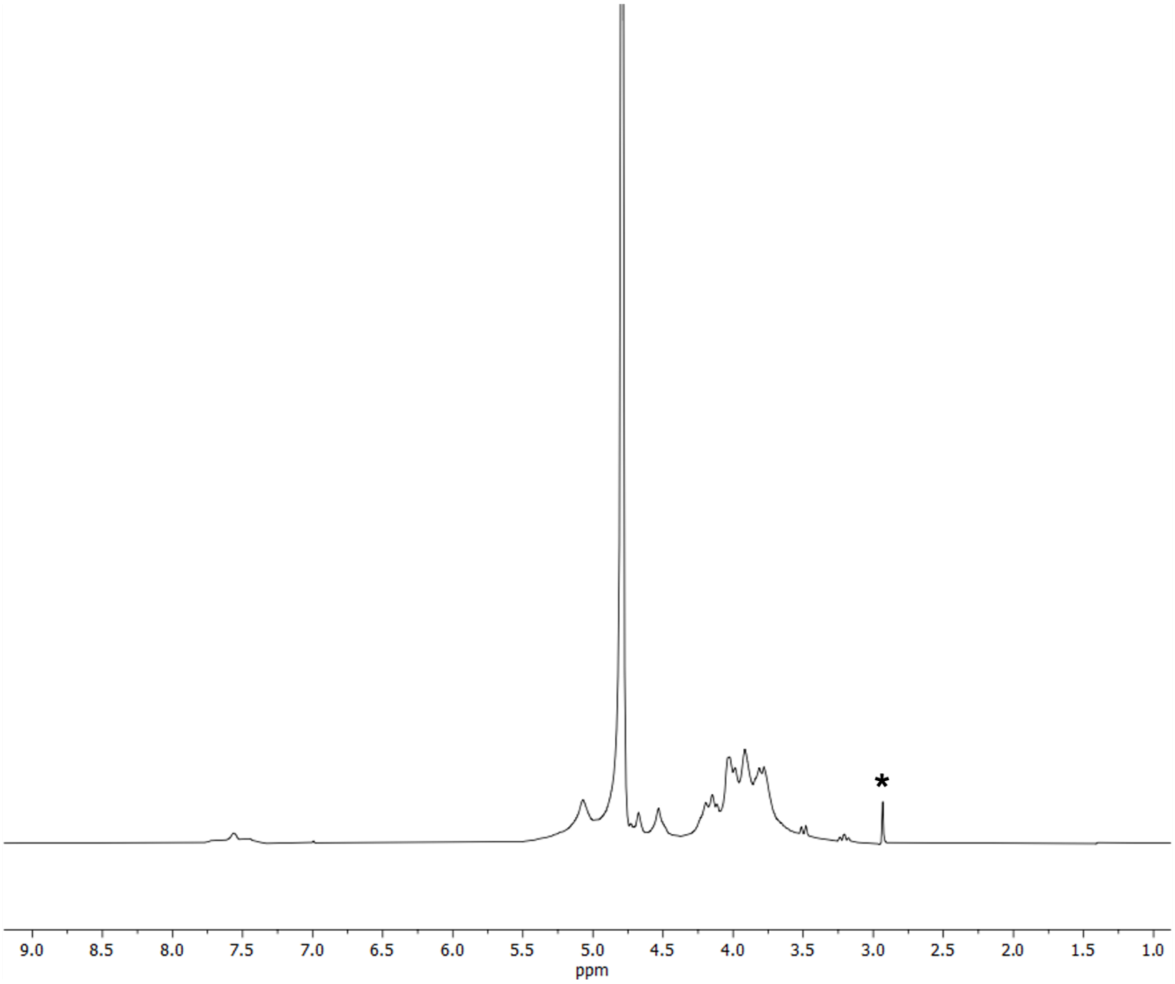

Supplementary Fig. 5. ^{1}H NMR spectrum (400 MHz, D_2O) of alginate functionalized with DBCO and FAM (Alg4-DBCO-FAM). * = DMSO.

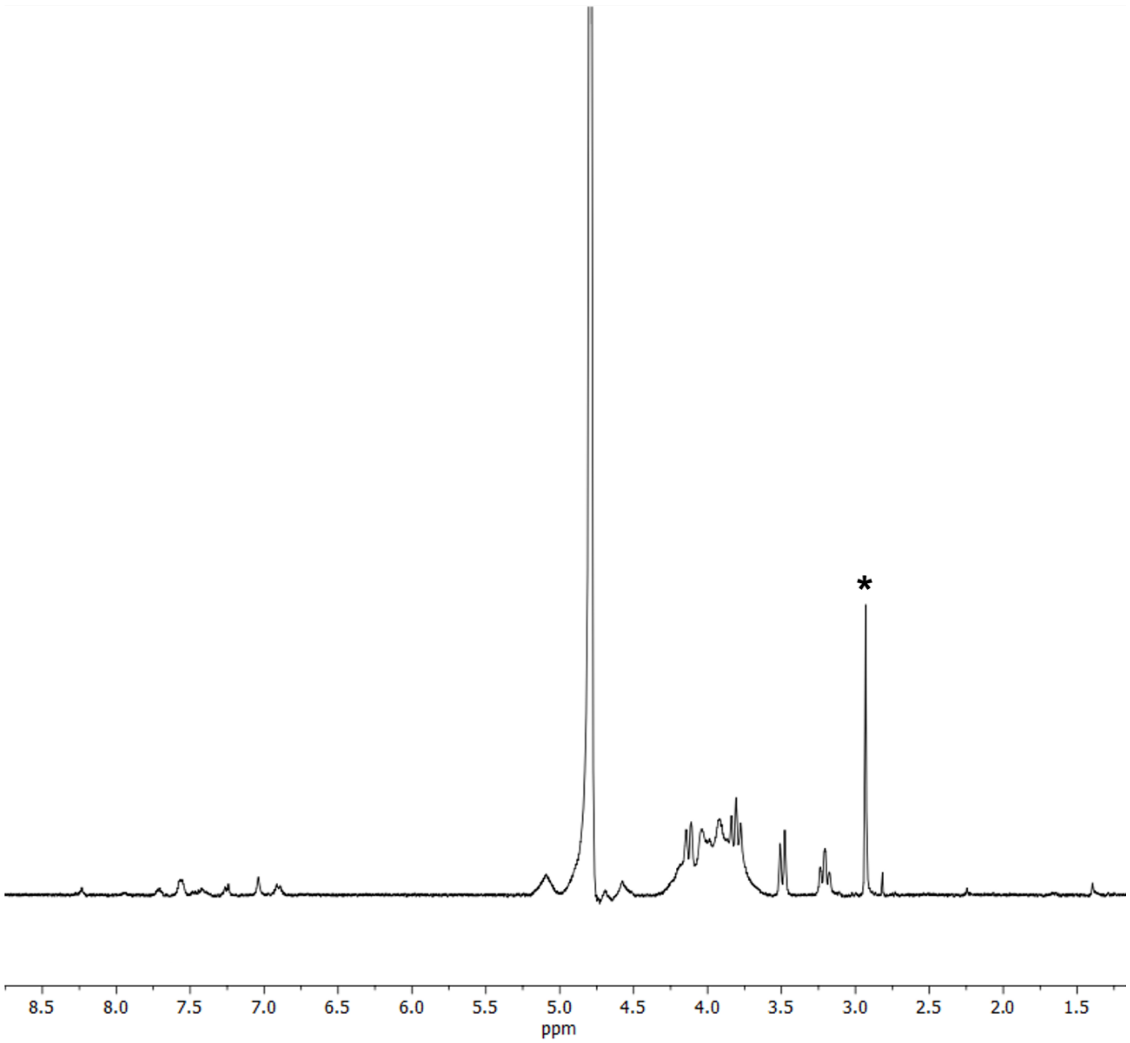

Supplementary Fig. 6. ^{1}H NMR spectrum (400 MHz, D_2O) of alginate functionalized with DBCO and FAM (Alg8-DBCO-FAM). * = DMSO.

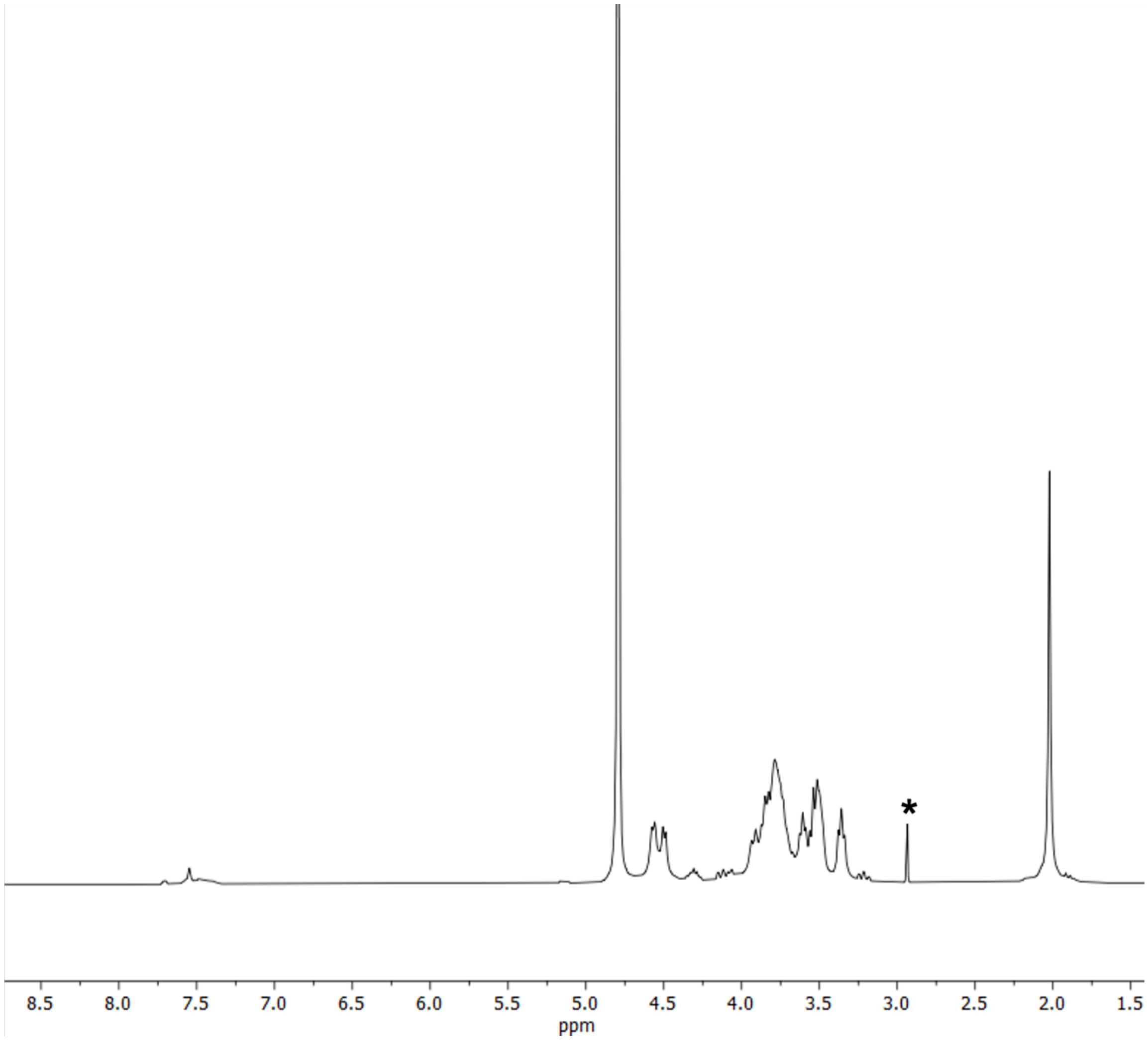

Supplementary Fig. 7. ^{1}H NMR spectrum (400 MHz, D_2O) of hyaluronic acid functionalized with DBCO and FAM (HA4-DBCO-FAM). * = DMSO.

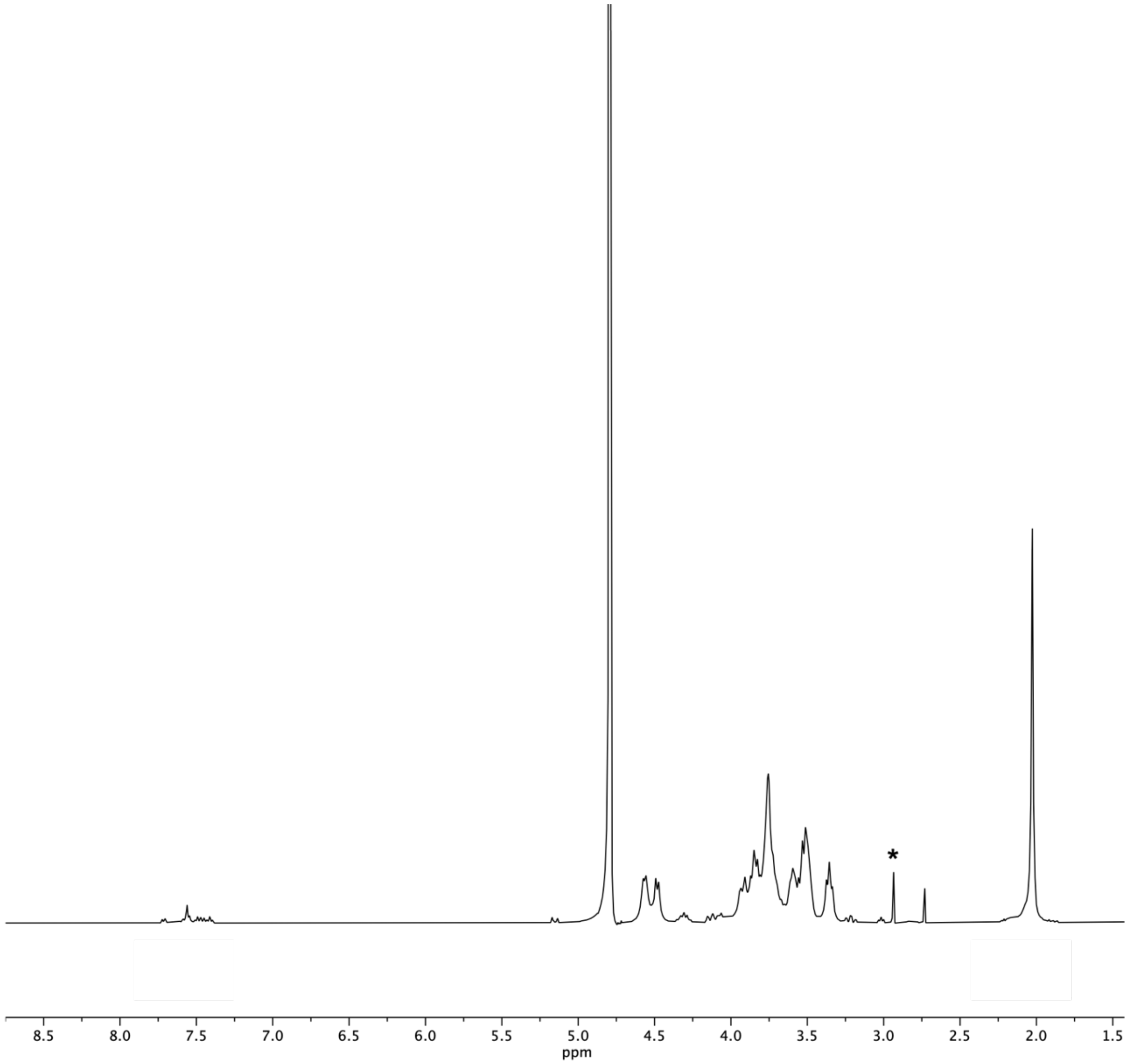

Supplementary Fig. 8. ^{1}H NMR spectrum (400 MHz, D_2O) of hyaluronic acid functionalized with DBCO and FAM (HA8-DBCO-FAM). * = DMSO.

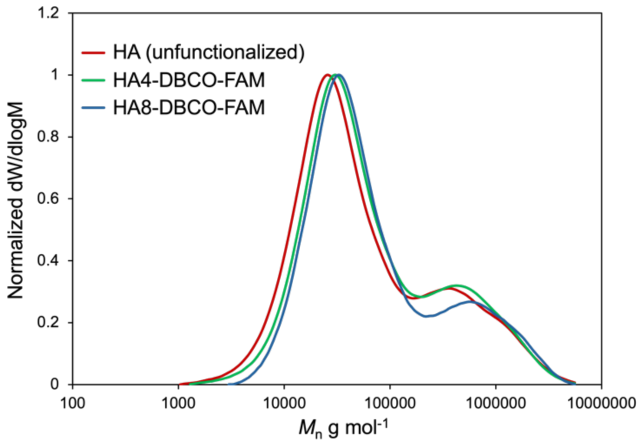

Supplementary Fig. 9. SEC of HA polymers (RI detection, elution with H_2O and 20% MeOH). PEG calibration.

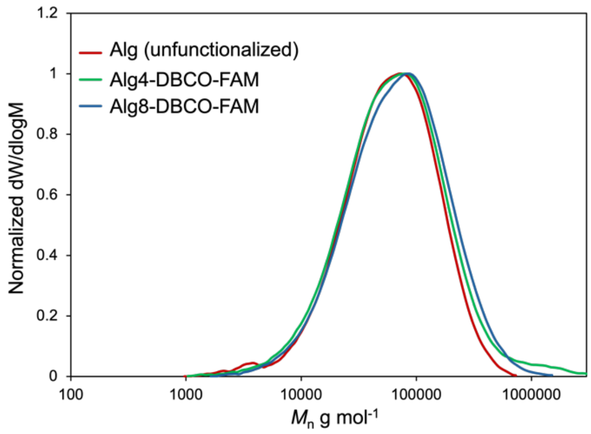

Supplementary Fig. 10. SEC of Alg polymers (RI detection, elution with H_2O and 20% MeOH). PEG calibration.

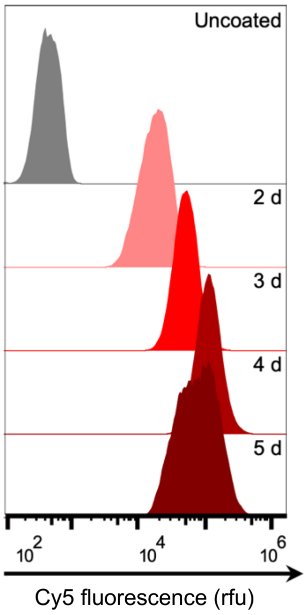

Supplementary Fig. 11. Flow cytometry graphs of HPCs treated with $Ac_4ManNAz$ for the indicated time periods (2 - 5 d) and incubated with Cy5-DBCO for 2 h.

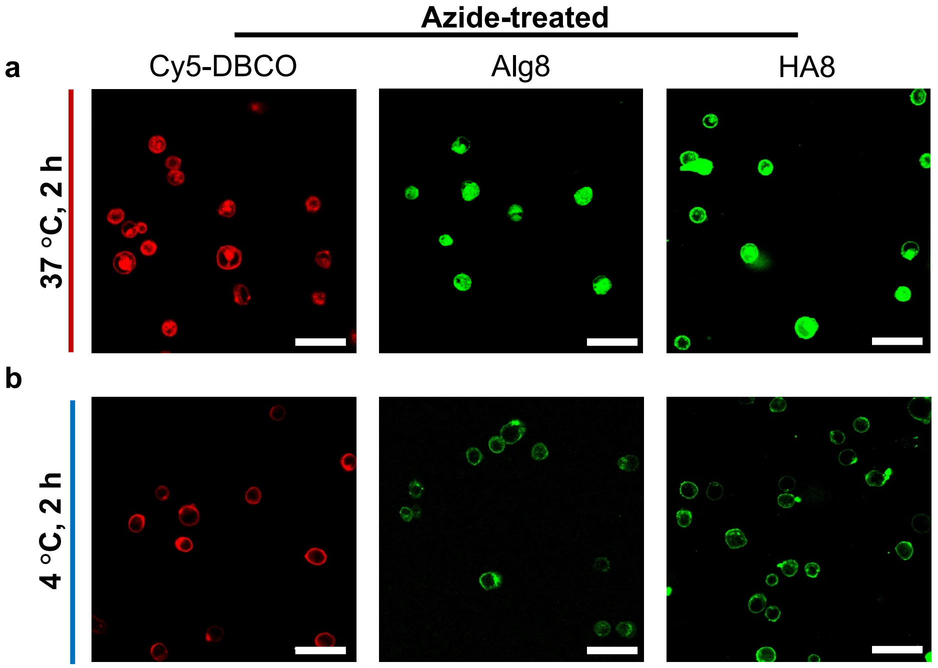

Supplementary Fig. 12. a. Representative confocal fluorescence microscopy images of azide-treated HPCs incubated at 37 °C with DBCO-Cy5, Alg8 and HA8 (left to right). **b.** Representative confocal fluorescence microscopy images of azide-treated HPCs incubated at 4 °C with DBCO-Cy5, HA8 and Alg8 (left to right). Scale bar = 50 μm.

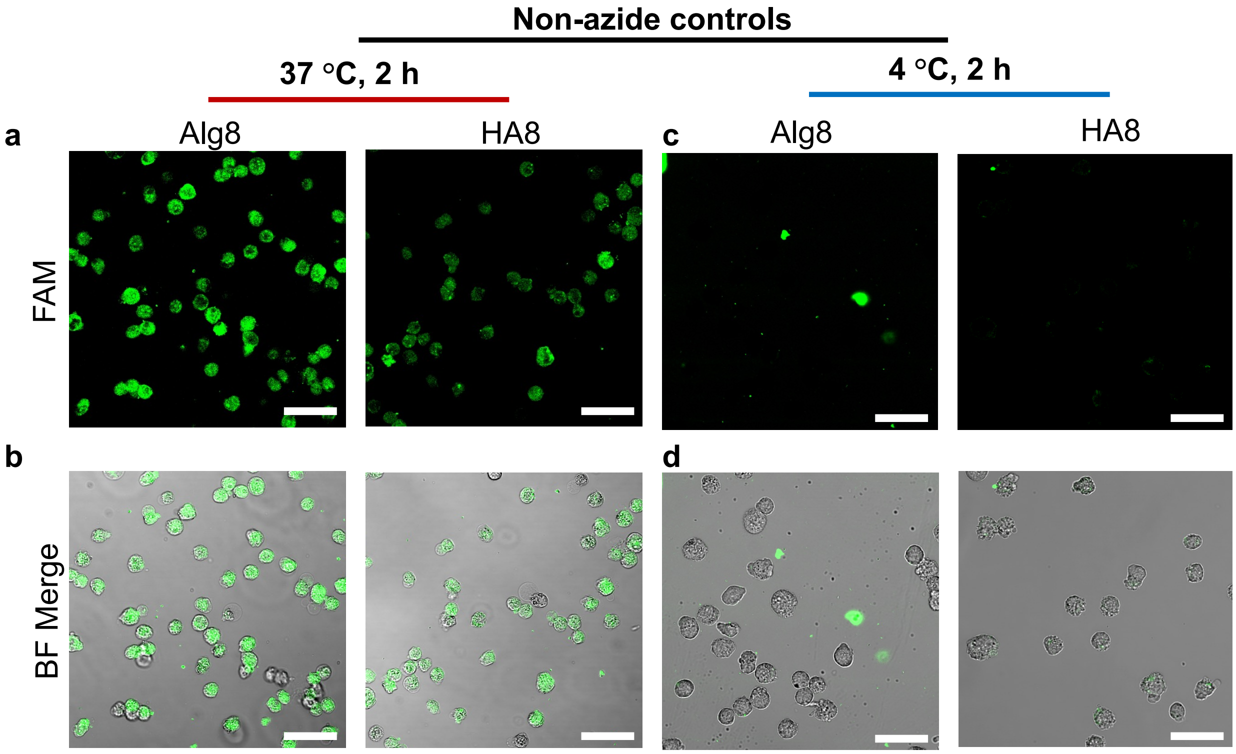

Supplementary Fig. 13 a. Representative confocal fluorescence microscopy images of HPCs which were not treated with $Ac_4ManNAz$ incubated at 37 °C with Alg8 (left) and HA8 (right). **b.** Fluorescence and brightfield merge of HPCs (not treated with azide) incubated at 37 °C with HA8 and Alg8 (left to right). **c.** Representative confocal fluorescence microscopy images of HPCs (not treated with azide) incubated at 4 °C with DBCO-Cy5, HA8 and Alg8 (left to right). **d.** Fluorescence and brightfield merge of HPCs (not treated with azide) incubated at 4 °C with HA8 and Alg8 (left to right). Scale bar = 50 µm.

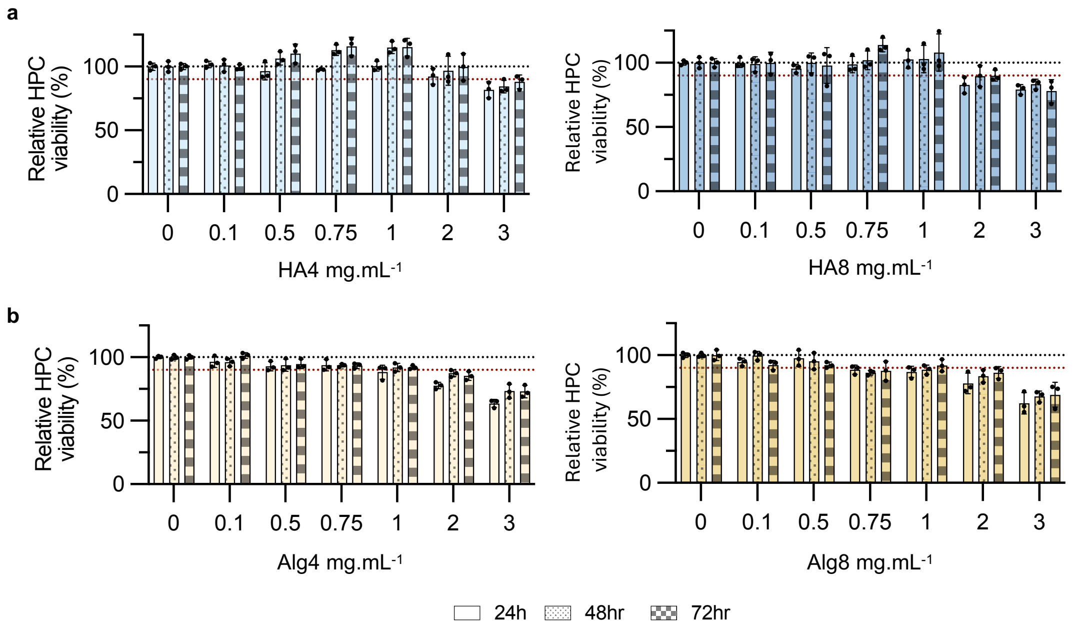

Supplementary Fig. 14. Viability of HPCs over 72 h after incubation with increasing concentrations (0 - 3 mg mL^{-1}) of DBCO-functionalized **a.** HA4, HA8, **b.** Alg4 and Alg8 (left to right, respectively) (n = 3 ± SD). Red and black-dotted lines indicate 90% and 100% cell viability, respectively, related to uncoated controls (0 mg mL^{-1}).

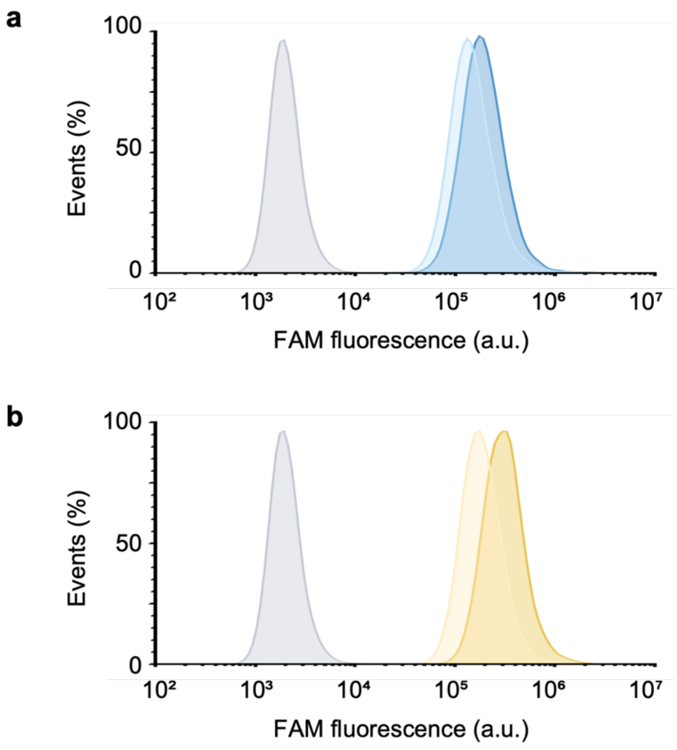

Supplementary Fig. 15. Flow cytometry graphs of HPCs uncoated (grey) and coated with HA4 and HA8 (a, light blue and dark blue respectively) and Alg4 and Alg8 (b, light yellow and dark yellow respectively).

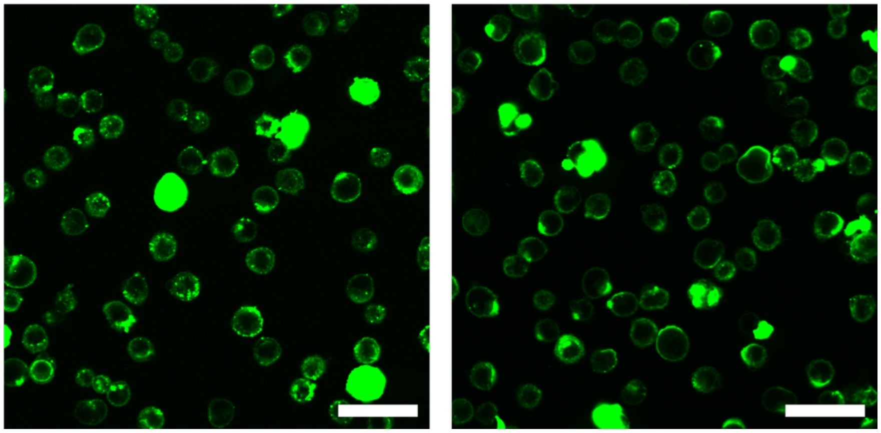

Supplementary Fig. 16. Representative confocal fluorescence microscopy images of Alg8 (left) and HA8 (right) coated cells after 2.5 h of incubation at 4 °C. Scale bar = 50 μm.

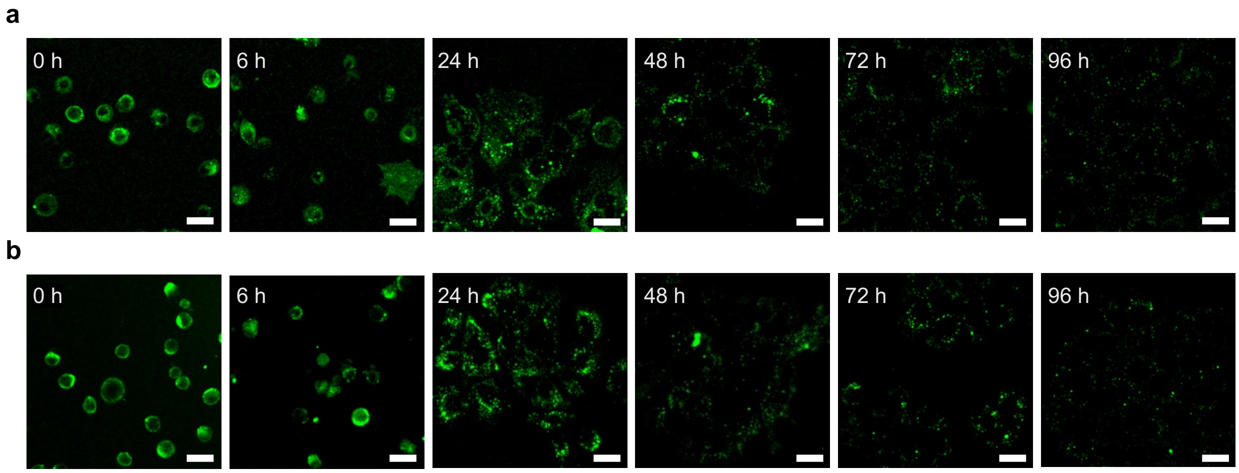

Supplementary Fig. 17. Confocal fluorescence microscopy images (Z-stacks) of HPCs in standard cell culture media after coating with HA4 (a) and Alg4 (b) captured over a period of 96 h. Scale bar = 20 μm.

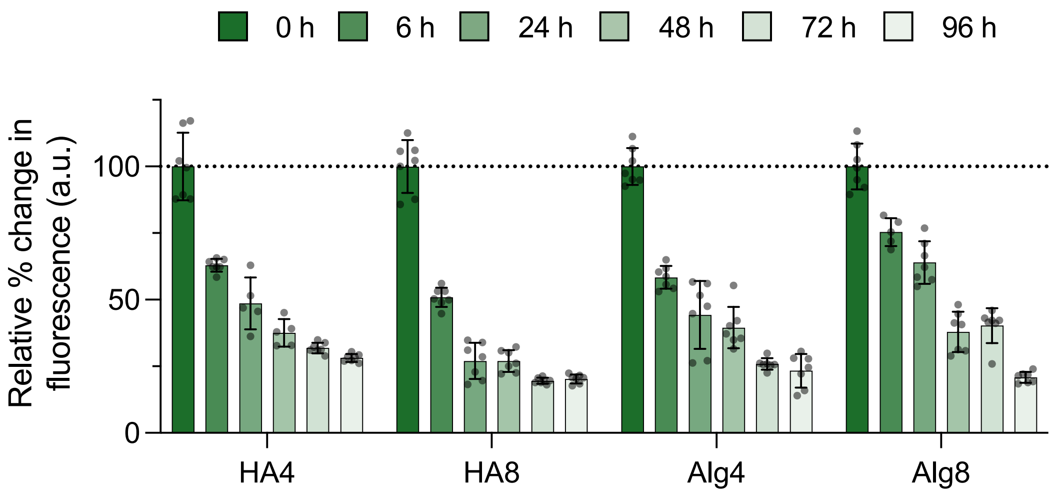

Supplementary Fig. 18. Graph showing the change in relative FAM fluorescence of coated HPCs over 4 days after plating onto Col-I. Fluorescence was quantified as a function of cell number per field area by analyzing captured confocal fluorescence microscopy Z-stack images using Olympus cellSens software (N = 3 ± SD). Dotted line represents average fluorescence intensities of HPCs directly after coating (0 h).

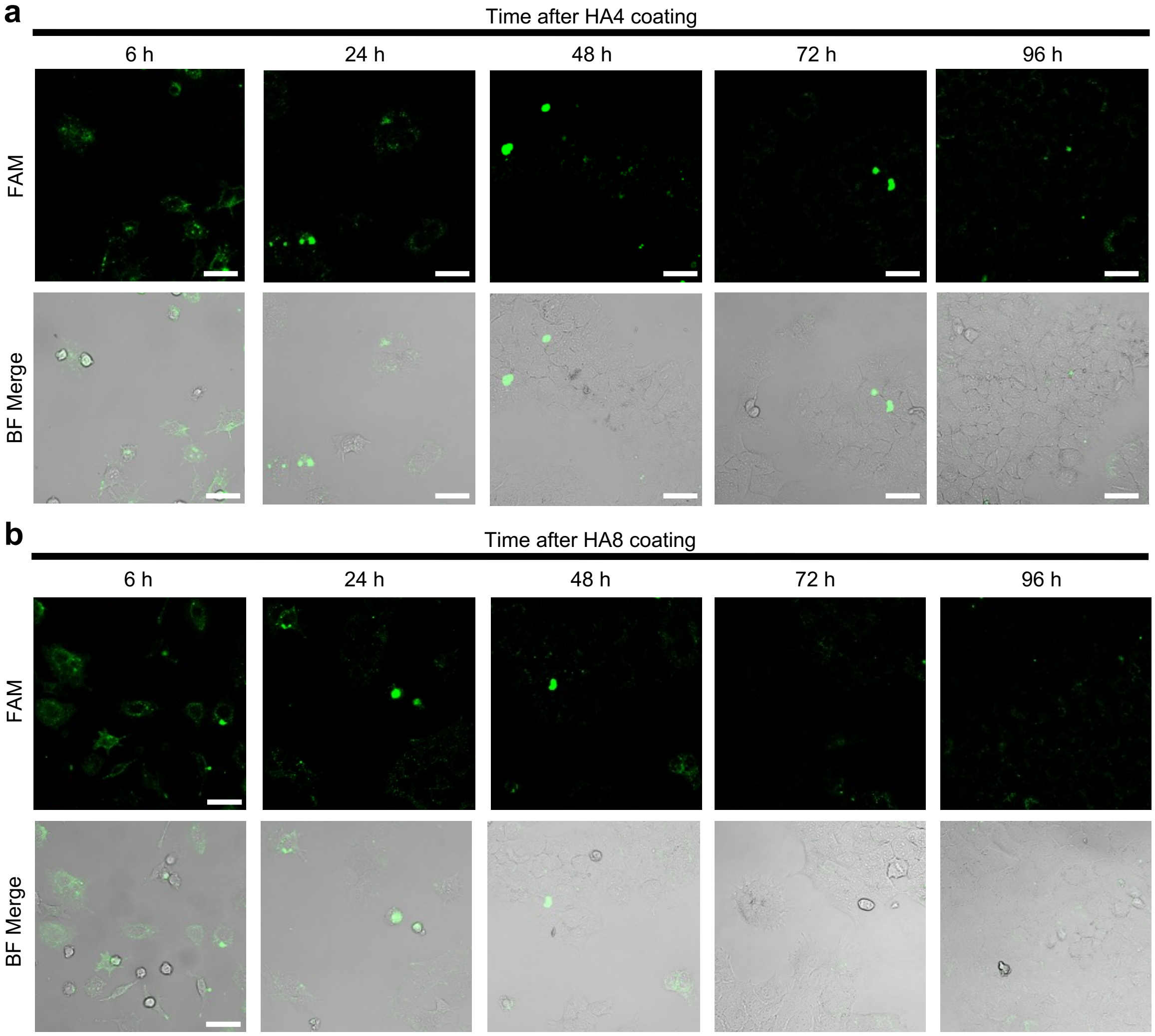

Supplementary Fig. 19. Confocal fluorescence microscopy images of HPCs (intracellular slices) in standard cell culture media after coating with HA4 (**a**) and HA8 (**b**) captured over a period of 96 h. Cells were incubated under standard conditions of 37 °C, 5% CO_2. BF = brightfield. Scale bar = 50 μm.

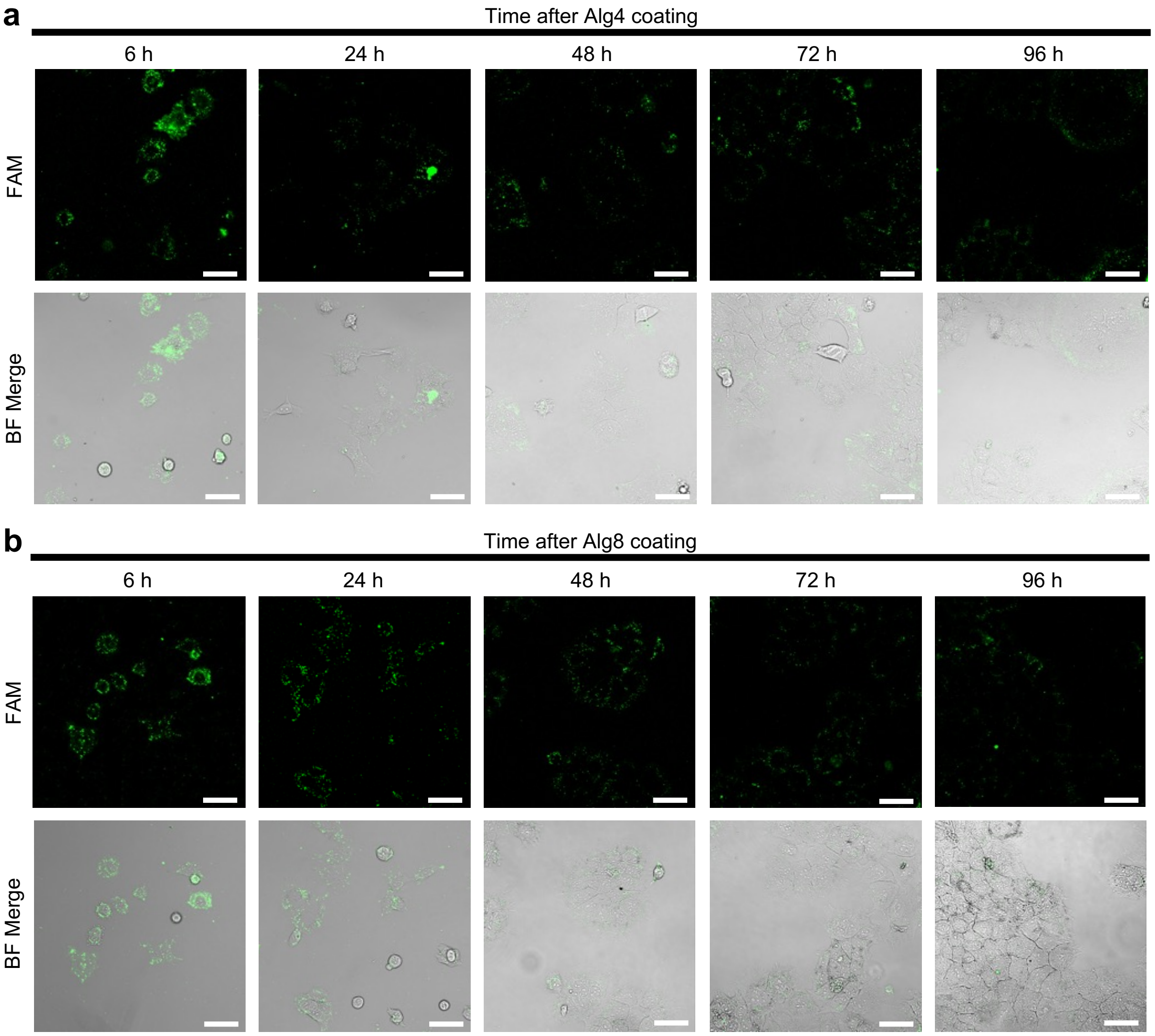

Supplementary Fig. 20. Confocal fluorescence microscopy images of HPCs (intracellular slices) in standard cell culture media after coating with Alg4 (**a**) and Alg8 (**b**) captured over a period of 96 h. Cells were incubated under standard conditions of 37 °C, 5% CO_2. BF = brightfield. Scale bar = 50 μm.

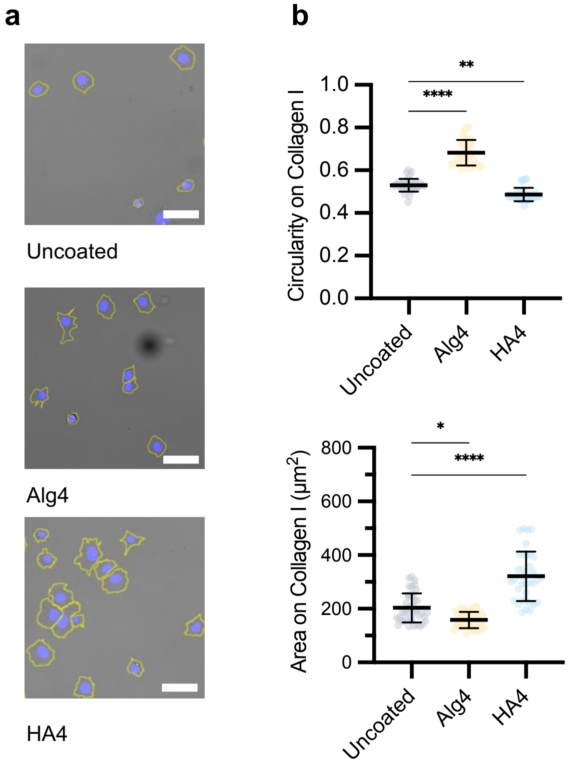

Supplementary Fig. 21. a. Representative confocal brightfield images of uncoated, Alg4, and HA4-coated HPCs left to adhere onto Col-I for 2 h. Nuclei are stained in blue **b.** Morphological parameters for area and circularity analyzed from confocal brightfield images for uncoated, Alg4, and HA4 coated HPCs after adhesion onto Col-I-coated plates. Scale bar = 50 µm (N = 3 ± SD, * P = <0.05, **** P = <0.0001).

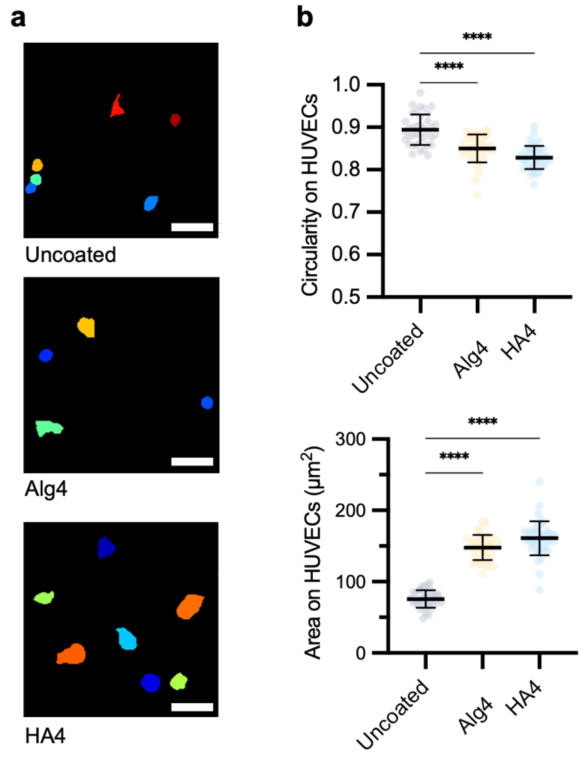

Supplementary Fig. 22. a. Representative confocal fluorescence microscopy image masks of uncoated, Alg4, and HA4-coated HPCs left to adhere onto HUVEC monolayers for 2 h. **b.** Morphological parameters for area and circularity analyzed form the image masks for uncoated, Alg4, and HA4 coated HPCs after adhesion to HUVEC monolayers. Scale bar = 50 µm (N = 3 ± SD, **** P = <0.0001).

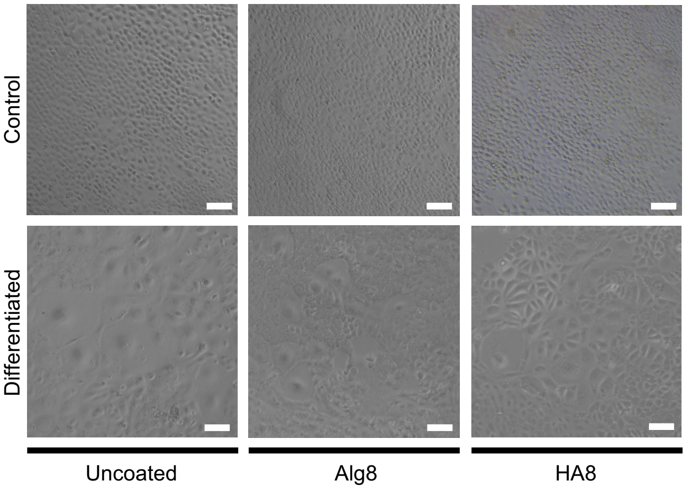

Supplementary Fig. 23. Phase-contrast microscopy images of HPCs after hepatocyte differentiation with murine Wnt3a. Scale bar = 20 μm.

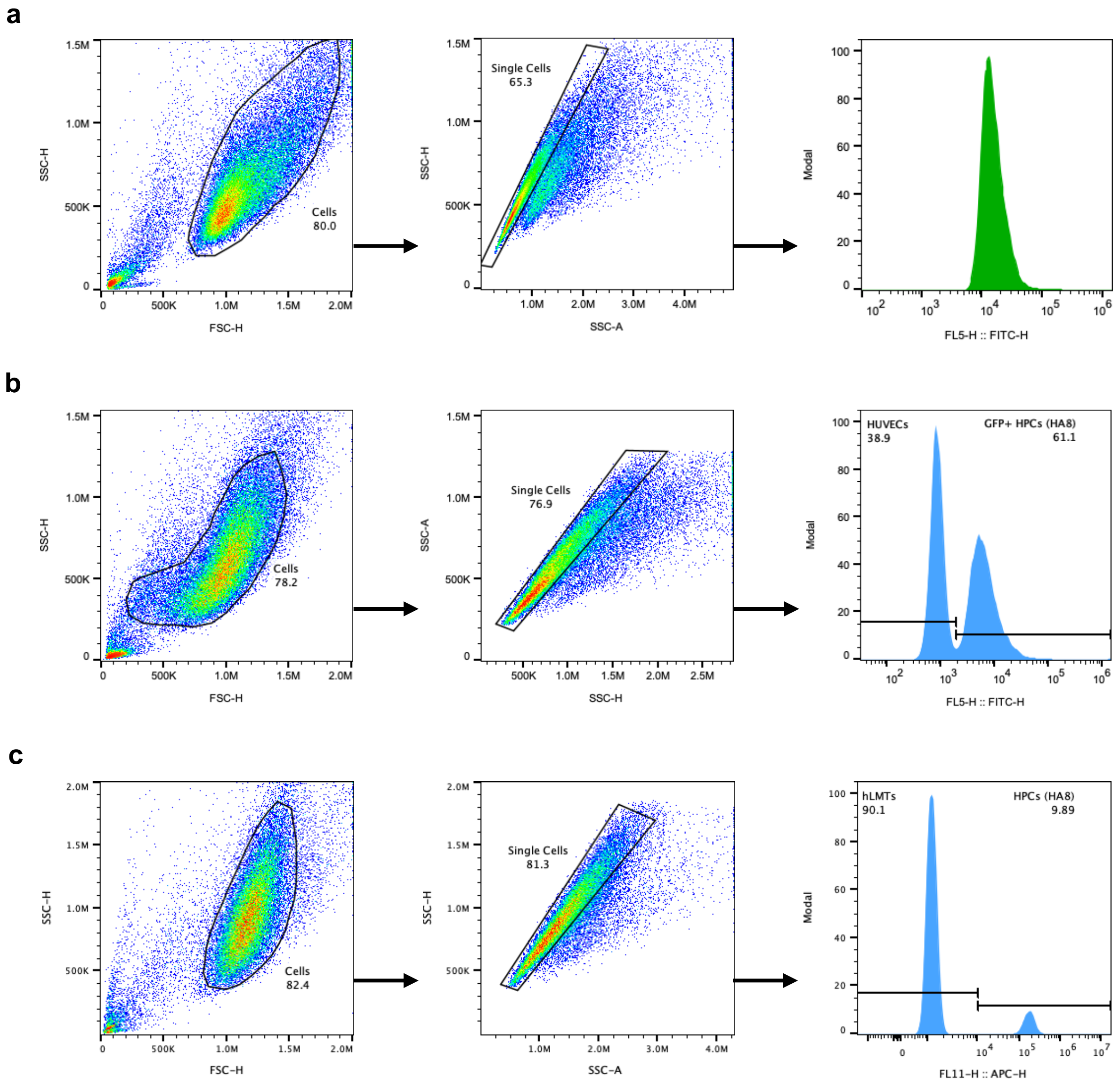

Supplementary Fig. 24. a. General gating strategy for cell debris and doublet discrimination in the fluorescence quantification of HPCs in Fig. 1c-d, Fig. 2c, and Supplementary Fig. 11 and 15. 2 h, HA4-coated HPCs from Fig. 1c were used as representative example. **b.** Gating strategy for the discrimination of HUVEC / HPC (GFP$^+$) cell populations. HA8 dataset from Fig. 3b was used as representative example. **c.** Gating strategy for the discrimination of hLMT / HPC (CellTracker DeepRed) populations. HA8 dataset from Fig. 4c was used as representative example.
