# Supplementary material for: Sweet and sticky: increased cell adhesion through click-mediated functionalization of regenerative liver progenitor cells
Source: Commun Biol. 2025 Jul 10;8:1023. doi: 10.1038/s42003-025-08408-x (PMC12246442; doi:10.1038/s42003-025-08408-x)
Supplement: Supplementary file 3 — Description of Additional Supplementary Materials [file 42003_2025_8408_MOESM3_ESM.pdf]

Description of Additional Supplementary Files

File name: Supplementary Data 1

Description: Source data for the main figures
