# Supplementary material for: Sweet and sticky: increased cell adhesion through click-mediated functionalization of regenerative liver progenitor cells
Source: Commun Biol. 2025 Jul 10;8:1023. doi: 10.1038/s42003-025-08408-x (PMC12246442; doi:10.1038/s42003-025-08408-x)
Supplement: Supplementary file 5 — Reporting Summary [file 42003_2025_8408_MOESM5_ESM.pdf]

nature portfolio

Corresponding author(s): Maria Chiara Arno
Last updated by author(s): Jun 10, 2025

Reporting Summary

Nature Portfolio wishes to improve the reproducibility of the work that we publish. This form provides structure for consistency and transparency in reporting. For further information on Nature Portfolio policies, see our Editorial Policies and the Editorial Policy Checklist.

Statistics

For all statistical analyses, confirm that the following items are present in the figure legend, table legend, main text, or Methods section.

n/a	Confirmed	
☐	☒	The exact sample size (*n*) for each experimental group/condition, given as a discrete number and unit of measurement
☐	☒	A statement on whether measurements were taken from distinct samples or whether the same sample was measured repeatedly
☐	☒	The statistical test(s) used AND whether they are one- or two-sided<br>*Only common tests should be described solely by name; describe more complex techniques in the Methods section.*
☒	☐	A description of all covariates tested
☒	☐	A description of any assumptions or corrections, such as tests of normality and adjustment for multiple comparisons
☐	☒	A full description of the statistical parameters including central tendency (e.g. means) or other basic estimates (e.g. regression coefficient) AND variation (e.g. standard deviation) or associated estimates of uncertainty (e.g. confidence intervals)
☐	☒	For null hypothesis testing, the test statistic (e.g. *F*, *t*, *r*) with confidence intervals, effect sizes, degrees of freedom and *P* value noted<br>*Give P values as exact values whenever suitable.*
☒	☐	For Bayesian analysis, information on the choice of priors and Markov chain Monte Carlo settings
☒	☐	For hierarchical and complex designs, identification of the appropriate level for tests and full reporting of outcomes
☒	☐	Estimates of effect sizes (e.g. Cohen's *d*, Pearson's *r*), indicating how they were calculated

Our web collection on statistics for biologists contains articles on many of the points above.

Software and code

Policy information about availability of computer code

Data collection	Beckman Coulter CytExpert (2.5.0.77) for acquiring flow cytometry data.<br>Olympus FV31S-SW (2.3.1) for acquisition of fluorescence images.<br>Microsoft Excel (Office 365) for raw data handing.<br>BMG Labtech Omega Control (3.35) for acquisition of data from FluoSTAR Omega microplate reader.
Data analysis	Olympus CellSens (1.18) for processing of confocal microscopy data.<br>FIJI (1.54f) for processing and analysing confocal microscopy data.<br>CellProfiler (4.2.6) for processing and analysing confocal microscopy data.<br>BMG Labtech Omega MARS (3.35) for processing and analysing plate reader measurements.<br>MestreNova (14.0) for processing and analysing NMR spectra.<br>FlowJo (10.10) for processing and analysing flow cytometry data.<br>Graphpad Prism (8.0.1) for processing, graphical plotting and statistical analysis.

For manuscripts utilizing custom algorithms or software that are central to the research but not yet described in published literature, software must be made available to editors and reviewers. We strongly encourage code deposition in a community repository (e.g. GitHub). See the Nature Portfolio guidelines for submitting code & software for further information.

Data

Policy information about availability of data

All manuscripts must include a data availability statement. This statement should provide the following information, where applicable:

- Accession codes, unique identifiers, or web links for publicly available datasets
- A description of any restrictions on data availability
- For clinical datasets or third party data, please ensure that the statement adheres to our policy

All data supporting the findings of this study are available within the paper and its Supplementary Information.

Research involving human participants, their data, or biological material

Policy information about studies with human participants or human data. See also policy information about sex, gender (identity/presentation), and sexual orientation and race, ethnicity and racism.

Reporting on sex and gender	n/a
Reporting on race, ethnicity, or other socially relevant groupings	n/a
Population characteristics	n/a
Recruitment	n/a
Ethics oversight	n/a

Note that full information on the approval of the study protocol must also be provided in the manuscript.

Field-specific reporting

Please select the one below that is the best fit for your research. If you are not sure, read the appropriate sections before making your selection.

☒ Life sciences ☐ Behavioural & social sciences ☐ Ecological, evolutionary & environmental sciences

For a reference copy of the document with all sections, see nature.com/documents/nr-reporting-summary-flat.pdf

Life sciences study design

All studies must disclose on these points even when the disclosure is negative.

Sample size	No statistical methods were used to pre-determine sample sizes. Multiple replicates were used for each experiment and representative images are shown in the microscopy views.
Data exclusions	Anomalous outliers were removed from morphological quantification data in Fig. 3, i.e. debris/artefacts which were wrongly segmented from the images.
Replication	All experimental conditions were evaluated across independent biological replicates at least three times. Similar and consistent behaviors were observed in all attempts carried out while reproducing the experiments concerning cell surface coatings and flow cytometry analysis of resulting fluorescences.
Randomization	Biological replicates were randomly allocated into experimental groups for the different coatings or time points. All images were acquired by taking randomly distributed fields of view across the entire field of cells. hLMTs were randomly assigned and distributed into the Akura ImmuneFlow chips, each allocated into experimental groups.
Blinding	The researchers were not blinded to the identities of the experimental conditions and measurement outcomes. All data analyses are based onobjectively measured data, and blinding would not affect these data values (e.g. biospecific adhesion and morphological measurements). All control samples were acquired and analysed under the same conditions as experimental samples and data analysis performed under the same software settings.

Reporting for specific materials, systems and methods

We require information from authors about some types of materials, experimental systems and methods used in many studies. Here, indicate whether each material, system or method listed is relevant to your study. If you are not sure if a list item applies to your research, read the appropriate section before selecting a response.

Materials & experimental systems

n/a	Involved in the study
☐	☒ Antibodies
☐	☒ Eukaryotic cell lines
☒	☐ Palaeontology and archaeology
☒	☐ Animals and other organisms
☒	☐ Clinical data
☒	☐ Dual use research of concern
☒	☐ Plants

Methods

n/a	Involved in the study
☒	☐ ChIP-seq
☐	☒ Flow cytometry
☒	☐ MRI-based neuroimaging

Antibodies

Antibodies used: Mouse Albumin ELISA kit (abcam, ab108792)

Validation: Albumin specific antibody pre-coated onto 96-well plates were validated using the provided Albumin kit standards.

Eukaryotic cell lines

Policy information about cell lines and Sex and Gender in Research

Cell line source(s): Primary cell line, derived from mouse. HPCs were provided as a kind gift from Dr Wei-Yu Lu (University of Edinburgh) following protocol published in http://dx.doi.org/10.1038/protex.2015.051.

Authentication: See http://dx.doi.org/10.1038/protex.2015.051.

Mycoplasma contamination: Cell lines tested negative for mycoplasma contamination.

Commonly misidentified lines (See ICLAC register): n/a

Plants

Seed stocks: n/a

Novel plant genotypes: n/a

Authentication: n/a

Flow Cytometry

Plots

Confirm that:

☒ The axis labels state the marker and fluorochrome used (e.g. CD4-FITC).

☒ The axis scales are clearly visible. Include numbers along axes only for bottom left plot of group (a 'group' is an analysis of identical markers).

☒ All plots are contour plots with outliers or pseudocolor plots.

☒ A numerical value for number of cells or percentage (with statistics) is provided.

Methodology

Sample preparation: Cells were suspended in PBS solution supplemented with 3% v/v fetal bovine serum and 3 mM EDTA and passed through a 40 µm cell strainer to ensure single cell analysis.

Instrument: Beckman Coulter CytoFLEX flow cytometer.

Software: CytExpert software (Beckman Coulter) was used for data collection. FlowJo v10.10 (BD) was used to analyse and plot data.

Cell population abundance	All sample measurements consisted of a minimum of 30,000 total recorded events.
Gating strategy	All flow cytometry data was collected by multiparameter analysis including FSC and SSC. Debris and doublets were gated out by determination of FSC/SSC patterns from uncoated controls. Voltage settings applied ensured that untreated control cells appeared at minimum fluorescence emission intensities (FITC and Cy5 channels) and to ensure fluorescence measurements were within the detection range (< 10^6 A.U.).

☒ Tick this box to confirm that a figure exemplifying the gating strategy is provided in the Supplementary Information.
